# Development of machine learning models to predict gestational diabetes risk in the first half of pregnancy

**DOI:** 10.1186/s12884-023-05766-4

**Published:** 2023-06-23

**Authors:** Gabriel Cubillos, Max Monckeberg, Alejandra Plaza, Maria Morgan, Pablo A. Estevez, Mahesh Choolani, Matthew W. Kemp, Sebastian E. Illanes, Claudio A. Perez

**Affiliations:** 1 Department of Electrical Engineering, Universidad de Chile, Av. Tupper 2007, 8370451 Santiago, Chile; 2 IMPACT, Center of Interventional Medicine for Precision and Advanced Cellular Therapy, Santiago, Chile; 3 Department of Obstetrics and Gynecology and Laboratory of Reproductive Biology, Faculty of Medicine, Universidad de los Andes, 7620001 Santiago, Chile; 4 Department of Obstetrics and Gynaecology, NUS Yong Loo Lin School of Medicine, National University of Singapore, 1E Kent Ridge Road, NUHS Tower Block, Level 12, Singapore, 119228 Singapore

**Keywords:** Gestational diabetes mellitus (GDM), GDM risk prediction, Machine learning models, Data augmentation, Widely available variables

## Abstract

**Background:**

Early prediction of Gestational Diabetes Mellitus (GDM) risk is of particular importance as it may enable more efficacious interventions and reduce cumulative injury to mother and fetus. The aim of this study is to develop machine learning (ML) models, for the early prediction of GDM using widely available variables, facilitating early intervention, and making possible to apply the prediction models in places where there is no access to more complex examinations.

**Methods:**

The dataset used in this study includes registries from 1,611 pregnancies. Twelve different ML models and their hyperparameters were optimized to achieve early and high prediction performance of GDM. A data augmentation method was used in training to improve prediction results. Three methods were used to select the most relevant variables for GDM prediction. After training, the models ranked with the highest Area under the Receiver Operating Characteristic Curve (AUCROC), were assessed on the validation set. Models with the best results were assessed in the test set as a measure of generalization performance.

**Results:**

Our method allows identifying many possible models for various levels of sensitivity and specificity. Four models achieved a high sensitivity of 0.82, a specificity in the range 0.72–0.74, accuracy between 0.73–0.75, and AUCROC of 0.81. These models required between 7 and 12 input variables. Another possible choice could be a model with sensitivity of 0.89 that requires just 5 variables reaching an accuracy of 0.65, a specificity of 0.62, and AUCROC of 0.82.

**Conclusions:**

The principal findings of our study are: Early prediction of GDM within early stages of pregnancy using regular examinations/exams; the development and optimization of twelve different ML models and their hyperparameters to achieve the highest prediction performance; a novel data augmentation method is proposed to allow reaching excellent GDM prediction results with various models.

**Supplementary Information:**

The online version contains supplementary material available at 10.1186/s12884-023-05766-4.

## Introduction

Gestational Diabetes Mellitus (GDM) is defined as any degree of glucose intolerance with onset or first recognition during pregnancy [1, 2]. In 2017, it was estimated that around 14% of pregnancies were affected by GDM worldwide [3]. The prevalence of GDM varies among countries and regions and is substantially impacted by the diagnostic criteria employed [3–6]. GDM is associated with increased risk of acute and chronic disease for both mother and developing fetus [1, 4, 7, 8]. Adverse fetal outcomes associated with GDM include increased risk of insulin resistance, macrosomia, preterm birth, respiratory distress, neonatal intensive care unit admission and stillbirth [9–11]. Adverse maternal outcomes associated with GDM include depression, a 7 to tenfold increase in the risk of developing Type 2 Diabetes Mellitus (T2DM; relative to non-GDM women), elevated risk of liver and renal disease, more adverse lipid profiles and a twofold increase in risk of cardiovascular disease [9–11], including insulin resistance.

There is no uniform consensus on the optimal diagnostic criteria for the diagnosis of GDM. The first diagnostic test for GDM recommended by O’Sullivan and Mahan in 1964 [12] employed a fasting three-hour oral glucose tolerance test (OGTT) using 100 g of glucose with whole-blood analyses, with two or more elevated measurements at fasting 3 h required for a GDM diagnosis [9]. A series of protocol amendments followed, leading to the development of a two-step protocol based around an initial screening test (1 h, non-fasting 50 g glucose challenge with cut-offs ranging from 130–140 mg/dl) followed by a diagnostic glucose tolerance test (measuring fasting, 1 h, 2 h, and 3 h glucose levels) [9, 12]. More recently, based on the finding of the Hyperglycemia and Adverse Pregnancy Outcome (HAPO) Study, a one-step screening strategy proposed by the International Association of Diabetes and Pregnancy Study Groups (IADPSG) recommended the use of a fasting two-hour 75 g oral glucose tolerance test [13]. Although the one-step IADPSG has the obvious advantage of requiring only a single test and one elevated glucose measurement, its use has raised concerns regarding GDM overdiagnosis [9]. Interestingly, several studies have reported that the prevalence of GDM as two to three-fold higher using the IADPSG one-step approach compared to the two-step screen and diagnose protocol, but no clear improvement in pregnancy outcomes. Highlighting the lack of consensus in the field, Fu and Retnakaran [9] note that although the one-step IADPSG protocol is endorsed by the International Federation of Gynecology and Obstetrics, the American Diabetes Association and the World Health Organization (WHO), the two-step screen and diagnose protocol is endorsed by the National Institutes of Health and the American College of Obstetricians and Gynecologists [9].

Irrespective of the diagnostic approach used, the current paradigm has a number of inherent disadvantages. OGTT is time consuming for clinicians and patients, it cannot easily be applied to the total population and is associated with a high false positive rate [14]. Results can be impacted strongly by pre-analytical laboratory practices; for example, room temperature glycolysis by leukocytes and erythrocytes prior to centrifugation can reduce glucose levels between five and seven percent per hour [15]; in a recent Australian study of 12,317 women, when centrifugation was performed within ten minutes of sample collection the GDM diagnosis rate nearly doubled from 11.6% to 20.6% using the IADPSG criteria [16]. Secondly, OGTT at 24–28 weeks of gestation does not facilitate treatment early in pregnancy. As articulated by Sweeting and colleagues [11], although most international guidelines recommend early antenatal GDM testing for high-risk mothers, there is no current consensus on testing approach or diagnostic thresholds [11]. Moreover, there is a lack of evidence to support improved pregnancy outcomes with the early diagnosis and treatment of GDM based on current approaches [11]. There is, however, evidence to show that a range of first trimester biomarkers can be used to predict GDM development later in pregnancy, and that fetal macrosomia can occur prior to a diagnosis of GDM being made [9]. What is clear, however, is the expectation that early and accurate prediction of GDM risk can lead to interventions that can help to better health outcomes for both mothers and babies [17–19].

### State of the art

With this objective in mind, several models have been developed to diagnose GDM during the early stages of gestation [20–35]. Some of these models use simple variables, such as age, previous GDM, a first-degree relative with a family history of diabetes, multiple pregnancies, fasting plasma glucose (FPG), glycated hemoglobin (HBA_1c_) and triglyceride [20]. A rapidly growing body of evidence shows that the application of machine learning (ML) to analyze data of this nature, and more general biophysical and socio-economic metrics (i.e., easily obtained from a patient history early in pregnancy) may allow a new means by which early and accurate predictions of GDM risk may be made [36]. Critically, such predictions may be able to be scaled to a population level as they do not require the taking of liquid biopsies, the administration of screening or diagnostic tests, and convey comparably little per-test cost. ML approaches have shown success in the prediction of preeclampsia [37], GDM from electronic health records [22], and pattern recognition [38]. In GDM prediction, various models have been used including Deep Neural Network (DNN) [20], Logistic Regression (LR) [21], Gradient Boosting [22], a LR and Extreme Gradient Boosting (XGBoost) [23], and Random Forest (RF) with LR [24]. A recent review [36] of ML-based models for the prediction of GDM before 24–28 weeks of pregnancy reported the viability of this approach to make predictions from general patient data, and emphasized the use of generic clinical variables. The best results of previously published models using similar input variables and GDM criterion are summarized in Table 8. Although several studies focusing on the prediction of GDM have been presented, a model that can reach high sensitivity and specificity for early prediction of GDM, and with the least number of variables, is still clinically needed. Additionally, variables that are widely available for screening examinations during pregnancy will allow a massive application of the prediction model, including low-income areas where more complex tests are not available, or may not be able to be executed in a highly standardized fashion (i.e., rigorous pre-analytical sample processing).

The main objective of our ML models is to predict the risk of developing GDM early in pregnancy in order to facilitate preventive treatment and reduce the risk of adverse maternal and fetal outcomes. As this was a retrospective study, all patients had OGTT data available for validation of the GDM diagnosis. It is worth noting that the OGTT was not used to develop the models but rather to validate the diagnosis of GDM. In the present submission we report the development of twelve different ML models, and the optimizing of their hyperparameters for the prediction of GDM, to achieve the highest classification performance, and the application of a variable selection process. Redundant data was eliminated to improve model performance.

## Materials and methods

### Database

**Fig. 1:**
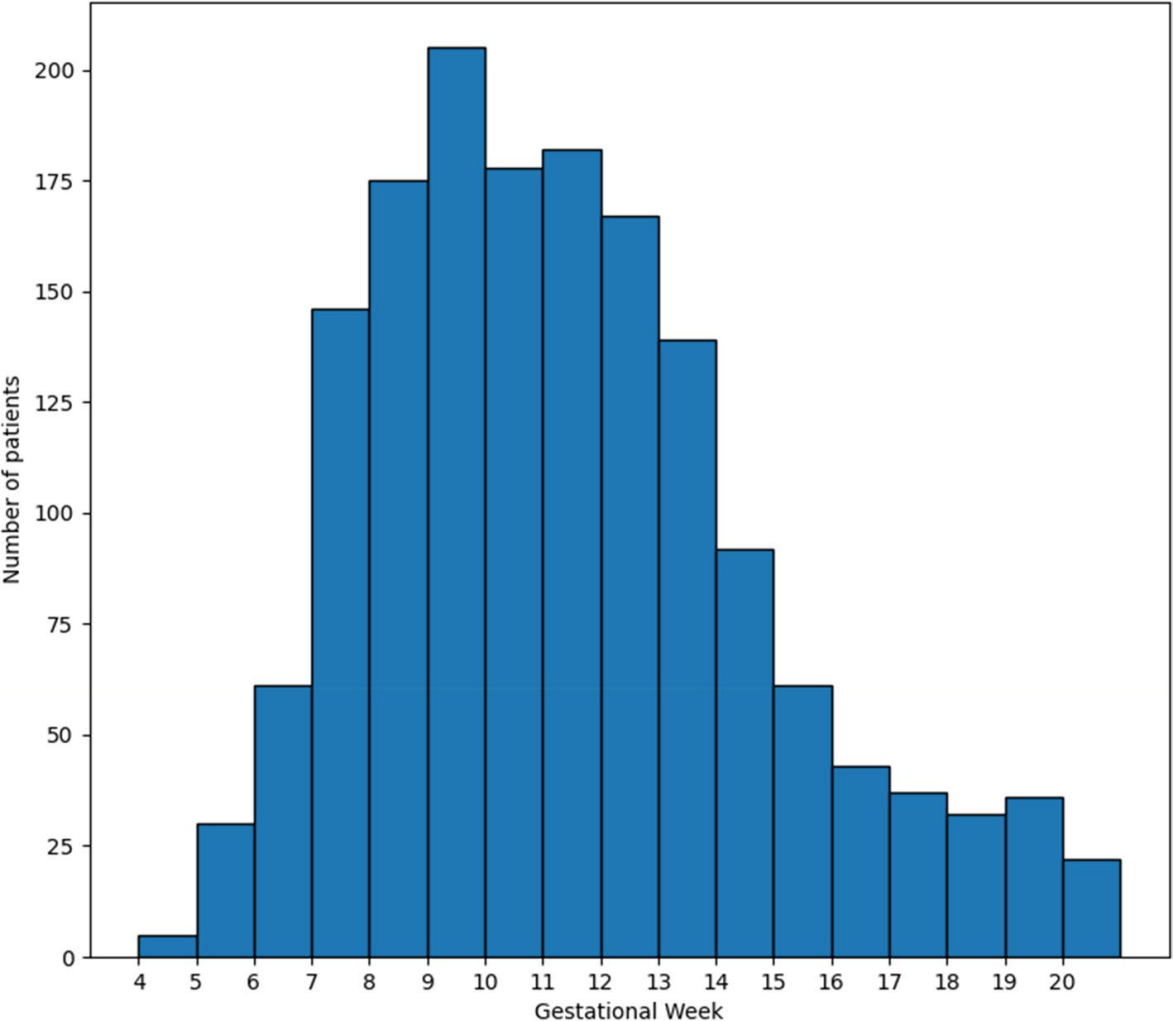
Histogram showing the number of first maternal visits per gestational week

The dataset used in this study was obtained from patients attending the Obstetrics and Fetal Medicine Unit of the Hospital Parroquial de San Bernardo, Santiago, Chile. The dataset included registries from 1,611 different pregnant patients, from 2019 to 2022. The patients included in the dataset have all the available variables/completed; patients with missing data are not included. A diagnosis of GDM was made using the IADPSG/HAPO criteria for gestational diabetes [13, 39], i.e., oral glucose tolerance test (75 g) fasting glycemia ≥ 92 mg/dl, or 2 h glycemia ≥ 153 mg/dl in the second trimester. Patients with Diabetes Mellitus that had been diagnosed before pregnancy were excluded from the dataset. Data was obtained during regular maternal visits at up to the 20th week of gestation. The third column of Table 1 shows the information on the variables and the gestational week at which the information was collected. Most of the data was obtained during the first maternal visit that happened anytime between the 4th and 20th weeks of pregnancy. We also added a histogram (Fig. 1) showing the number of patients per gestational week for the first maternal visit. As in previous work [20, 22, 24, 27, 28, 30, 32, 35], our study was retrospective and therefore the dataset was available as described. Patients with Diabetes Mellitus diagnosed before pregnancy were excluded from the dataset. The data for the input to the model of each continuous variable was normalized (by subtracting the average and dividing it by the standard deviation), e.g., age, weight, height, and Body Mass Index (BMI) at the first visit, and the first trimester fasting glucose level. The database was divided into three partitions: training set (70%), validation set (10%), and testing set (20%).

**Table 1:** Clinical variables of the patients. IQR, interquartile range

Variable/ Feature	Non-GDM women (*n* = 1,382) Mean (IQR)	GDM women (*n* = 229) Mean (IQR)	Acquisition (GW)
Age	27.64 (23–32)	31.11 (27–36)	4–20
Pregnancy Type	1.01 (1–1)	1.02 (1–1)	4–20
Maternal Weight (first control) [kg]	71.62 (60–81)	81.77 (69–92)	4–20
Height [m]	1.59 (1.55–1.63)	1.59 (1.55–1.63)	4–20
BMI (Body Mass Index) (first control)	28.18 (24.03–31.64)	32.17 (28.16–35.83)	4–20
Gravidity	1.24 (0–2)	1.69 (0–2)	4–20
Parity	1.02 (0–2)	1.38 (0–2)	4–20
Abortions	0.22 (0–0)	0.32 (0–0)	4–20
Vaginal deliveries	0.79 (0–1)	1.03 (0–2)	4–20
Caesarean deliveries	0.22 (0–0)	0.34 (0–1)	4–20
Stillbirths	0.01	0.03	4–20
First trimester fasting glycemia [mg/dL] (1TFG)	77.22 (72–83)	87.12 (80–93)	4–12
OGTT (fasting) [mg/dL]	74.28 (69–81)	95.48 (86–101)	24–28
OGTT (2 h) [mg/dL]	99.39 (84–114)	142.87 (120–171)	24–28
	**(%)**	**(%)**	
Tobacco	7.74	11.79	4–20
Alcohol	3.62	4.80	4–20
Illicit Drugs	2.89	0.87	4–20
Cardiac Disease	0.65	0.44	4–20
Biliary Disease	1.01	2.18	4–20
Urinary Tract Disease	2.32	4.80	4–20
Chronic kidney Disease	0.36	0.00	4–20
Inflammatory bowel Disease	0.07	0.44	4–20
Chronic lung diseases	2.31	3.05	4–20
Systemic lupus erythematosus /Antiphospholipid antibody syndrome	0.14	0.44	4–20
Psychiatric Disorders	1.88	3.49	4–20
Endocrine Disorders	0.36	0.87	4–20
Gynecological Disorders	3.40	7.42	4–20
Epilepsy	1.09	0.44	4–20
Insulin Resistance	2.46	6.99	4–20
Hypothyroidism	4.05	9.17	4–20
Chronic Hypertension	4.70	12.66	4–20
Antihypertensive Drugs	3.55	10.04	4–20

### Data augmentation

Data augmentation (DA) is a common method used in ML to improve training results [40, 41]. We generated a DA method on the training set adapted to the diagnosis of GDM by restricting the data values within physiological ranges for each input. The ranges for the creation of new data were given by a specialist in Obstetrics/Gynecology. The DA approach was used to create new patients for training the models based on the original patients, changing some input values slightly as follows: i) Age: Newly created patients must be in a range of ± 2 years compared to the original ones; ii) First Trimester Glycemia Test: New created patients must be in a range of ± 5 mg/dL only if the original patient has a result between 66 and 94 mg/dL, or over 105 mg/dL in this test; iii) Height: Newly created patients must be in a range of ± 3 cm compared to the original ones; iv) Weight: Newly created patients must be in a range of ± 5 kg compared to the original ones; and v) BMI: The BMI was adapted to the changes of height and weight in the newly created patients. A new patient should not be created if the new BMI classification was different from that of the original patient. We used the BMI classification proposed by the WHO [42].

For the experiments we also considered a limited range for the DA range of values provided by a medical specialist. The original and the limited range values are shown in Table 2. Several cases for DA were determined by increasing the number of cases in the training set to generate a total number of cases reaching values of 120%, 140%, 160%, 180% and 200%, relative to the original number of cases, which was 100%.

**Table 2:** Data augmentation (DA) range of values provided by the medical specialist, and a limited range of values both are used for the experiments

DA\Columns	Age (Years)	1TFG (mg/dL)	Height (cm)	Weight (kg)	BMI
Expert original range	± 2	± 5	± 3	± 5	*
Limited Expert range	± 1	± 1	± 1	± 2	*

1TFG (First Trimester Fasting Glycemia Test)

^*^ The BMI value is computed according to the new values in height and weight. However, the new patient is created only if the classification of the BMI of the new patient is the same as that of the original patient. We use the classification, proposed by the WHO, that has also been used by other organizations [42]

### Prediction models

Twelve different ML models and their hyperparameters were optimized to achieve the highest prediction performance. Gaussian Naïve Bayes (GNB) and Bernoulli Naïve Bayes (BNB), Decision Trees (DT), Support Vector Machines (SVMs), Multi-Layer Perceptron (MLP), K-Nearest Neighbors (KNN), Logistic Regression (LR), Random Forest (RF), Extra Trees (ET) [43, 44], Balanced Random Forest (BRF) [45], Gradient Boosting (GB), implemented in Extreme Gradient Boosting (XGB) [46], and Light Gradient Boosting Machine (LGBM) [47] approaches were used. All the models were trained with the training set computing over 3,000 combinations of hyperparameters. For example, for the SVM, various types of kernels were used; for the MLP, different combinations of layers and solver were used; for the models based on Trees, various types of “criteria” were used; and for ensemble, different numbers of estimators were employed, among many other hyperparameters.

### Model implementation and hyperparameters

The models were implemented in Python 3.9.12 using Scikit-Learn [43], Imbalanced-Learn [45], XGBoost [46], and LGBM [47] libraries. The main hyperparameters used for each model are: GNB “var_smoothing” [43]; BNB”alpha” [44]; DT”criterion”,”max_depth”, “max_leaf_nodes”, “splitter” [43]; SVM “kernel”, “degree”, “decision_function_shape”, “C” [43]; MLP “solver”, “hidden_layer_sizes”, “activation”, “learning_rate_init”, “max_iter”, “learning_rate”, “early_stopping” [43]; KNN “algorithm”, “leaf_size”, “p”, “n_neighbors” [43]; LR”C”,”solver” [43], RF, ET and BRF “n_estimators”, “criterion” [43, 45]; XGB “n_estimators”, “eta”, “booster”, “gamma”, “max_depth” [46]; LGBM “n_estimators”, “boosting_type”, “learning_rate” [47].

Table 3 shows all the hyperparameters that were used in the Grid Search, and the range of values analyzed.

**Table 3:** Hyperparameters used in each model type

Hyperparameter	Used by	Ranges [lower bound, upped bound]
“var_smoothing”	Gaussian Naïve Bayes	[1e-10, 1e-7]
“alpha”	Bernoulli Naïve Bayes	[1e-10, 1]
“criterion”	Decision Tree, Random Forest, Extra Trees, Balanced Random Forest	“gini”, “entropy”
“max_depth”	Decision Tree, Extreme Gradient Boosting	[1, 20]
“max_leaf_nodes”	Decision Tree	[6, 384]
“splitter”	Decision Tree	“best”, “random”
“kernel”	SVM	“linear”, “poly”, “rbf”, “sigmoid”
“degree”	SVM	[1, 3]
“decision_function_shape”	SVM	“ovo”, “ovr”
“C”	SVM, Logistic Regression	[0.0001, 10]
“solver”	Multi-Layer Perceptron	“sgd”, “adam”
“hidden_layer_sizes”	Multi-Layer Perceptron	[8, 256], hidden layers: [1, 10]
“activation”	Multi-Layer Perceptron	“logistic”, “tanh”, “relu”
“learning_rate_init”	Multi-Layer Perceptron	[0.001, 0.1]
“max_iter	Multi-Layer Perceptron	20000
“early_stopping”	Multi-Layer Perceptron	True, False
“learning_rate”	Multi-Layer Perceptron	“constant”, “invscaling”, “adaptive”
“algorithm”	K-Nearest Neighbors	“auto”, “ball_tree”, “kd_tree”, “brute”
“leaf_size”	K-Nearest Neighbors	[1, 30]
“p”	K-Nearest Neighbors	[1, 4]
“n_neighbors”	K-Nearest Neighbors	[1, 25]
“solver”	Logistic Regression	“newton-cg”, “lbfgs”, “liblinear”, “sag”, “saga”
“n_estimators”	Random Forest, Extra Trees, Balanced Random Forest, Extreme Gradient Boosting, Light Gradient Boosting Machine	[10, 2000]
“eta”	Extreme Gradient Boosting	[0.001, 0.3]
“booster”	Extreme Gradient Boosting	“gbtree”, “gblinear”, “dart”
“gamma”	Extreme Gradient Boosting	[0, 1]
“boosting”	Light Gradient Boosting Machine	“gbdt”, “rf”, “dart”, “goss”
“learning_rate”	Light Gradient Boosting Machine	[0.001, 0.1]

### Model evaluation

The results obtained with the combination of hyperparameters values were assessed in a fivefold cross validation (CV) [48] using data from the training set and performing a grid search on the hyperparameter values. Grid search allows finding near optimal values for the hyperparameters via multiple evaluations of various combinations for each one. An input selection [49] was performed to select the best variables to be used in the prediction task to improve the model results and reduce input redundant variables to each model. The input variable selection was performed using 3 methods: F-test of ANOVA (Analysis of Variance), Chi-Square Test, and Mutual Information (also known as Information Gain) [43]. The models were trained, evaluated, and tested with various combinations of input variables selected by these 3 methods. After adjustment with the training set, the top 15% of the models ranked with the highest area under the ROC curve, AUCROC [50], were selected and assessed on the validation set. Models with the best results on the validation set were selected to obtain a good balance between high Sensitivity and good Specificity [50]. Finally, the selected models were assessed in the test set as a measure of generalization performance. The test set was not used in any previous step involving training or selection of the best models. Models were also trained using DA on the training and validation sets, but no DA was performed on the test set. The best results were chosen using sensitivity and specificity as the main metrics of performance. The accuracy, sensitivity, specificity and recall macro are measured with a specific decision threshold, calculated by using the validation dataset to determine this threshold. The ROC curve is created based on the different decision thresholds that modify sensitivity, also known as True Positive Rate (TPR), as a function of the false positive (FP). The formulas are the following: Accuracy = *(TP* + *TN)/(TP* + *FP* + *TN* + *FN)*, Sensitivity = *TP/(TP* + *FN)*, Specificity = *TN/(TN* + *FP)*, Recall Macro = *(Sensitivity* + *Specificity)/2.*

## Results

### Population characteristics

A total of 1,611 pregnant women were included in this study. The database was partitioned into 1,127 cases for the training set, 161 in the validation set, and 323 (39 positive of GDM) were part of the test set. The prevalence of GDM was 14.21% (229/1,611). The input variables to the models are described in Table 1.

### Variable selection

The most relevant 12 variables selected by the 3 methods: F-Test ANOVA, Chi-Square, and Mutual Information, are displayed on Table 4.

**Table 4:** The most relevant twelve variables for GDM prediction were selected by using four methods: F-Test ANOVA, Chi-Square, Mutual Information and BRF

Ranking	F-Test ANOVA	Chi-Square	Mutual Information	BRF
1	1TFG	1TFG	1TFG	1TFG
2	BMI	Maternal Weight	BMI	BMI
3	Maternal Weight	BMI	Age	Maternal Weight
4	Age	Age	Antihypertensive Drugs	Age
5	Chronic Hypertension	Gravidity	Maternal Weight	Height
6	Gravidity	Chronic Hypertension	Inflammatory Bowel Disease	Gravidity
7	Antihypertensive Drugs	Parity	Illicit Drugs	Parity
8	Parity	Antihypertensive Drugs	Chronic Kidney Disease	Vaginal Deliveries
9	Insulin Resistance	Abortions	Urinary Tract Disease	Abortions
10	Hypothyroidism	Vaginal Deliveries	Insulin Resistance	Cesarean Deliveries
11	Vaginal Deliveries	Insulin Resistance	Psychiatric Disorders	Hypothyroidism
12	Abortions	Hypothyroidism	Cardiac Disease	Chronic Hypertension

We selected the most important variables (features) in the dataset by removing irrelevant or redundant variables. This allows us to have a small number of variables which is useful for a clinical application. The methods used for this purpose are commonly employed in ML (F-test of ANOVA, Chi-Square Test, and Mutual Information). This variable selection also avoids the overfitting problem and achieves improved performance compared to that of using all the features [49]. For example, variables such as Pregnancy Type or Stillbirth are not selected by the variable selection methods, but may decrease the performance of models such as Multi-Layer Perceptron. Additionally, one of the models used to select variables was the BRF (see Table 4). The ranking obtained with a nonlinear model, BRF, is similar to those obtained with statistical methods, confirming that these are the relevant variables.

### Model performance

Table 5 shows the model type, number of input variables, whether or not DA was used, with “w/o DA” meaning that Data Augmentation is not used in this model, “DA LE”, meaning Data Augmentation w/Limited Expert range, “DA EO”, meaning Data Augmentation w/Expert original range, and the results of the following: Accuracy, Sensitivity, Specificity, Recall Macro, AUCROC, False Positives (FP), False Negatives (FN), and FP + FN. Table 5 show the top 4 models for each sensitivity level with the model that has the highest AUCROC in bold type, for models with up to 12 variables. All these metrics were computed for each model in the test set. As mentioned in the Methods section, the test set was only used to test the generalization capacity of the models. The test set was not used to train or to select the hyperparameters of the models. On Table 5 we show the results of models that reached a sensitivity above 0.9231 in the test set (model numbers 1 to 16), while model numbers 17 to 36 show the results of models with sensitivity above 0.7949 but below 0.9231 in the test set. Models with high sensitivity allow minimizing FN when screening patients. Sensitivity is important since the main goal is to prevent the serious consequences of GDM that may occur in mothers and babies even several years after pregnancy. Our method allows identifying many possible models for various levels of sensitivity and specificity. For example, model numbers 29–32 on Table 5 all have a high sensitivity of 0.82 and a specificity in the range 0.72–0.74, with accuracy between 0.73–0.75; AUCROC of 0.81; and Recall Macro between 0.77 and 0.78. A model could be selected from these ranges to have a good compromise between low numbers of FN and FP as is shown in the last column of Table 5.

**Table 5:** Top four models for different sensitivity levels, sensitivity ≥ 0.9231 (model number 1 to 16) and with sensitivity < 0.9231 and ≥ 0.7949 (model number 17 to 36), and up to 12 variables

Model	Model Type	Number of input variables	Data Augmentation	Accuracy	Sensitivity	Specificity	Recall Macro	AUC ROC	FP	FN	FP + FN
**1**	**MLP**	**12**	**w/o DA**	**0.3994**	**1**	**0.3169**	**0.6585**	**0.8189**	**194**	**0**	**194**
2	MLP	10	DA EO	0.3715	1	0.2852	0.6426	0.7741	203	0	203
3	MLP	11	DA LE	0.3715	1	0.2852	0.6426	0.7890	203	0	203
4	MLP	11	DA LE	0.3653	1	0.2782	0.6391	0.7874	205	0	205
**5**	**MLP**	**8**	**DA LE**	**0.5511**	**0.9744**	**0.4930**	**0.7337**	**0.8002**	**144**	**1**	**145**
6	SVM	5	DA LE	0.5480	0.9744	0.4894	0.7319	0.8161	145	1	146
7	SVM	5	DA LE	0.5480	0.9744	0.4894	0.7319	0.8161	145	1	146
8	MLP	4	DA EO	0.5387	0.9744	0.4789	0.7266	0.8052	148	1	149
**9**	**SVM**	**5**	**DA EO**	**0.6068**	**0.9487**	**0.5599**	**0.7543**	**0.8234**	**125**	**2**	**127**
10	MLP	4	DA EO	0.5759	0.9487	0.5246	0.7367	0.8159	135	2	137
11	MLP	3	w/o DA	0.5728	0.9487	0.5211	0.7349	0.8165	136	2	138
12	MLP	4	DA LE	0.5728	0.9487	0.5211	0.7349	0.8082	136	2	138
**13**	**SVM**	**5**	**DA EO**	**0.6130**	**0.9231**	**0.5704**	**0.7468**	**0.8234**	**122**	**3**	**125**
14	MLP	6	w/o DA	0.6006	0.9231	0.5563	0.7397	0.8221	126	3	129
15	MLP	8	DA EO	0.6006	0.9231	0.5563	0.7397	0.8183	126	3	129
16	LR	3	DA EO	0.6006	0.9231	0.5563	0.7397	0.8159	126	3	129
**17**	**MLP**	**5**	**DA LE**	**0.6594**	**0.8974**	**0.6268**	**0.7621**	**0.8199**	**106**	**4**	**110**
18	MLP	5	w/o DA	0.6594	0.8974	0.6268	0.7621	0.8146	106	4	110
19	MLP	5	DA LE	0.6563	0.8974	0.6232	0.7603	0.8178	107	4	111
20	MLP	7	DA LE	0.6563	0.8974	0.6232	0.7603	0.8118	107	4	111
**21**	**MLP**	**7**	**DA LE**	**0.6873**	**0.8718**	**0.6620**	**0.7669**	**0.8160**	**96**	**5**	**101**
22	MLP	10	DA LE	0.6811	0.8718	0.6549	0.7634	0.8078	98	5	103
23	MLP	9	DA LE	0.6780	0.8718	0.6514	0.7616	0.8137	99	5	104
24	MLP	9	DA EO	0.6749	0.8718	0.6479	0.7598	0.8137	100	5	105
**25**	**MLP**	**6**	**DA LE**	**0.7090**	**0.8462**	**0.6901**	**0.7681**	**0.8142**	**88**	**6**	**94**
26	MLP	9	DA EO	0.7090	0.8462	0.6901	0.7681	0.8022	88	6	94
27	MLP	10	w/o DA	0.7028	0.8462	0.6831	0.7646	0.8063	90	6	96
28	MLP	9	DA EO	0.7028	0.8462	0.6831	0.7646	0.8022	90	6	96
**29**	**SVM**	**12**	**w/o DA**	**0.7554**	**0.8205**	**0.7465**	**0.7835**	**0.8135**	**72**	**7**	**79**
30	SVM	12	w/o DA	0.7461	0.8205	0.7359	0.7782	0.8135	75	7	82
31	SVM	7	DA LE	0.7399	0.8205	0.7289	0.7747	0.8143	77	7	84
32	SVM	7	DA LE	0.7368	0.8205	0.7254	0.7729	0.8143	78	7	85
**33**	**SVM**	**7**	**DA LE**	**0.7399**	**0.7949**	**0.7324**	**0.7636**	**0.8143**	**76**	**8**	**84**
34	SVM	10	DA LE	0.7337	0.7949	0.7254	0.7601	0.8173	78	8	86
35	MLP	5	DA EO	0.7276	0.7949	0.7183	0.7566	0.8120	80	8	88
36	MLP	9	DA EO	0.7245	0.7949	0.7148	0.7548	0.8068	81	8	89

Abbreviations: *w/o DA* No data augmentation, *DA LE* Data augmentation w/limited expert range, *DA EO* Data augmentation w/expert original range

Note: The best model for each sensitivity level is in bold typeface

**Fig. 2:**
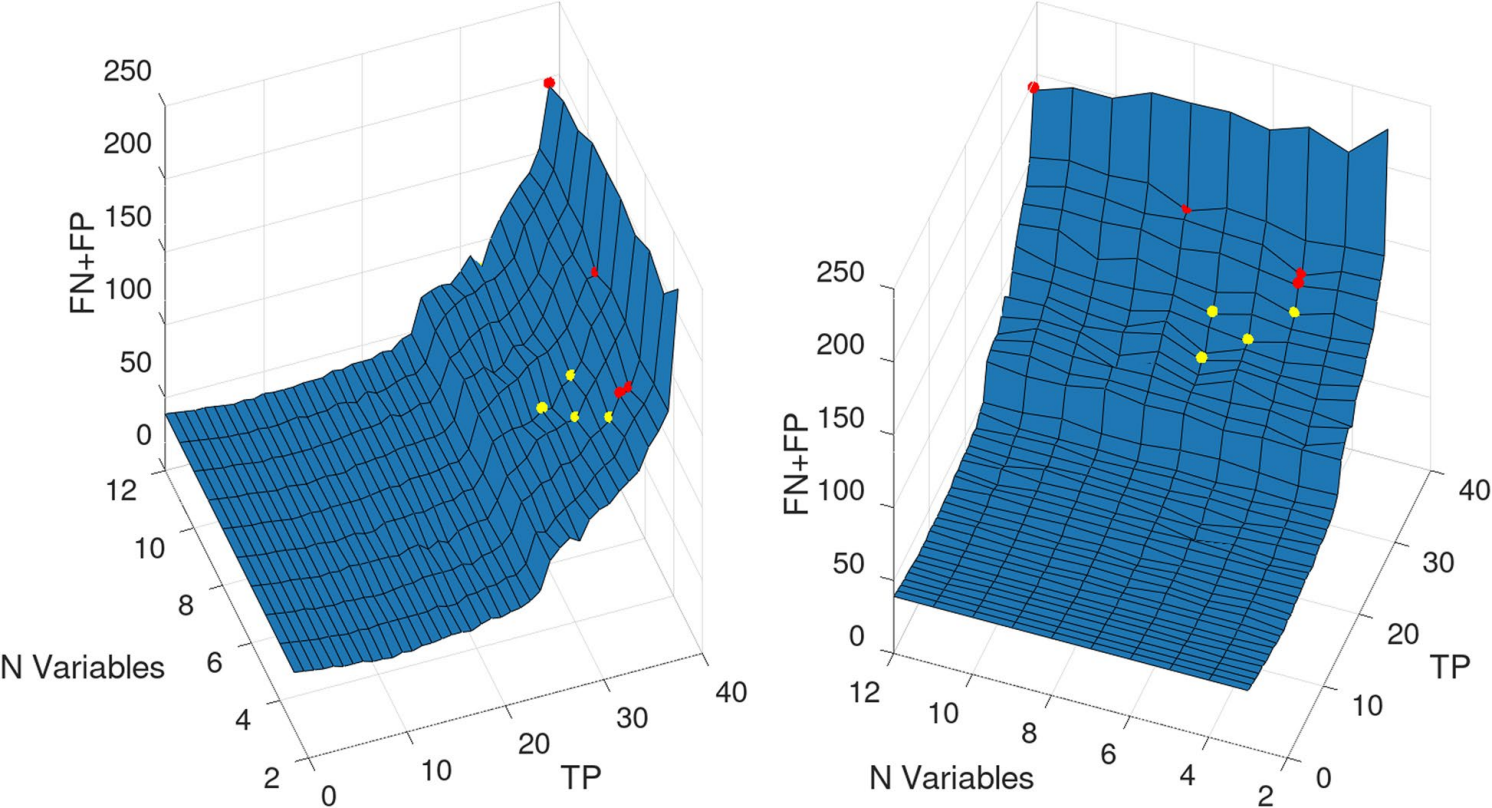
Surface with all models available, including various values of hyperparameters, for various levels of error (FP + FN), True Positives, and number of variables. The red dots represent the best models in bold type from Table 5 with sensitivity above 0.9231 (model numbers 1, 5, 9, and 13), and the yellow dots represent the best models from Table 5 with sensitivity above 0.7949 but below 0.9231 (model numbers 17, 21, 25, 29, and 33)

Another possible choice of model could be model 17 (Table 5) with sensitivity of 0.89 that requires just 5 variables (1TFG, Age, BMI, Maternal Weight, and Gravidity). This model reaches an accuracy of 0.65, a specificity of 0.62, Recall Macro of 0.76, and AUCROC of 0.82. Models 17–20 reach the same sensitivity of 0.89 with small changes in accuracy, specificity, Recall Macro and AUCROC. The best models for sensitivity 0.89 are all MLPs. It can be seen on Table 5, and on Fig. 2 that there are several choices of models for predicting various levels of sensitivity, with a trade-off on specificity.

Figure 2 shows two different views of the same surface plotting the model results for various values of the total number of errors (FP + FN), True Positives, and number of input variables for each model. Several choices of the models are available for reaching high sensitivity (low FN), and high specificity (low FP) with a small number of input variables. On the surface plotted in Fig. 2 the red dots represent the best models in bold type from Table 5 with sensitivity above 0.92 (model numbers 1, 5, 9, and 13), and the yellow dots represent the best models from Table 5 with sensitivity above 0.79 but below 0.92 (model numbers 17, 21, 25, 29, and 33).

**Fig. 3:**
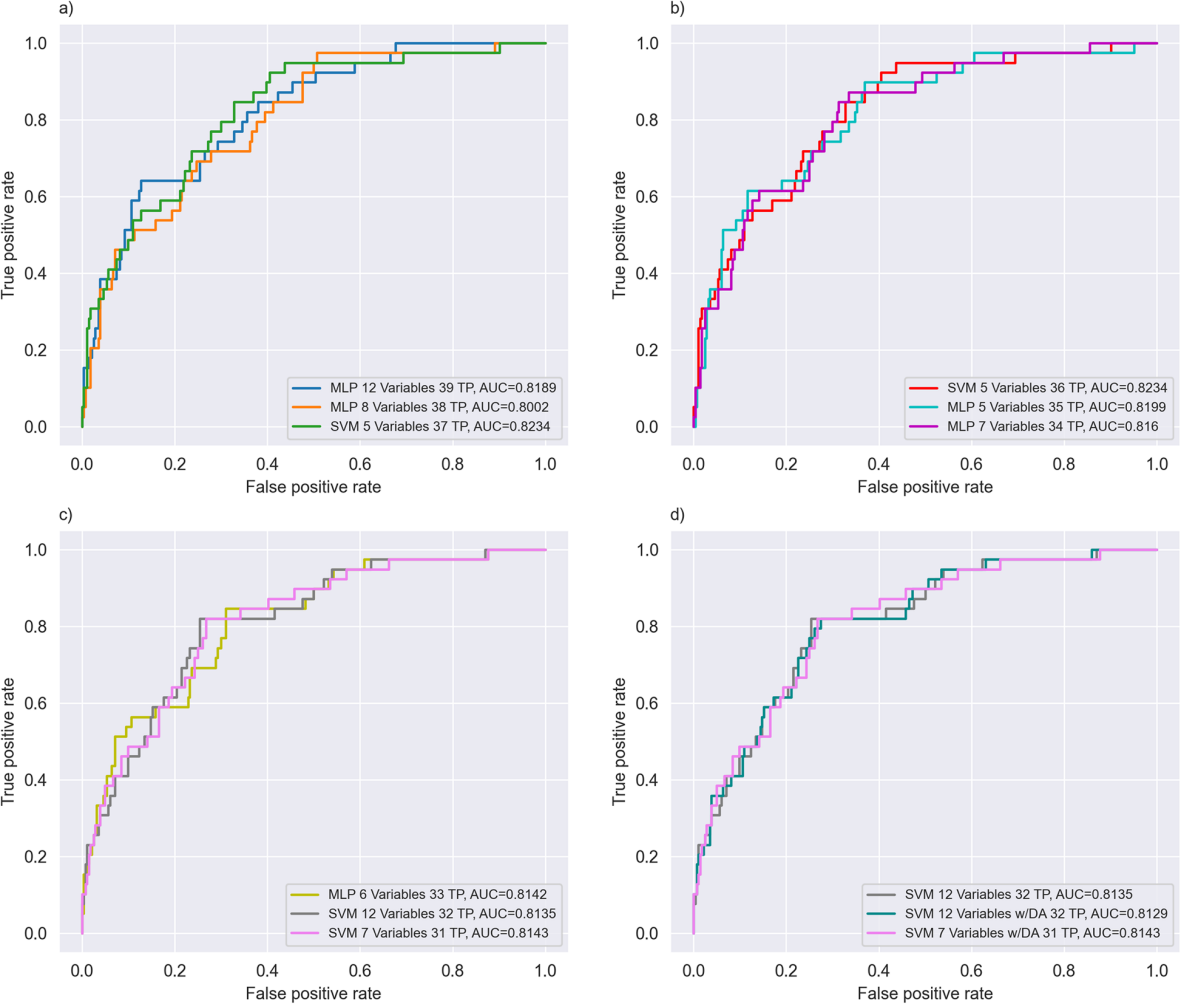
**a** ROC curves of the best models with sensitivities of 1 (MLP 12 variables), 0.9744 (MLP 8 variables), and 0.9487 (SVM 5 variables). **b** ROC curves of the best models with sensitivities of 0.9231 (SVM 5 variables), 0.8974 (MLP 5 variables), and 0.8718 (MLP 7 variables). **c** ROC curves of the best models with sensitivities equal to 0.8462 (MLP 6 variables), 0.8205 (SVM 12 variables), and 0.7949 (SVM 7 variables). **d** ROC curve for model number 29 in Table 5 with the best Recall Macro (gray, SVM 12 variables), and a comparison of the same model with Data Augmentation (cyan), and a model with a lower number of variables (pink, SVM 7 variables)

Figure 3 shows the ROC curves for each of the 9 best models with a fixed sensitivity starting at sensitivity of 1 (a), up to a sensitivity 0.79 (d). These best models for each sensitivity level appear in bold type in Table 5. Figure 3(a) shows the ROC curves for the best models with sensitivities of 1, 0.9744 and 0.9487. Figure 3(b) shows the ROC curves of the best models with sensitivities of 0.9231, 0.8974 and 0.8718. Figure 3(c) shows the ROC curves of the best models with sensitivities of 0.84, 0.82, and 0.79. Finally, Fig. 3(d) shows the ROC curves for model number 29 in Table 5 with the best recall macro (gray), and a comparison with the same model having DA (cyan), and the same model with a lower number of variables (pink). This model, number 29, has the lowest number of FP + FN.

Table 6 shows the best models for different sensitivity levels, with more than 12 input variables. It can be observed that models 38, 42, 43 and 45 reached a slightly better FP + FN than our best selected models shown on Table 5. Nevertheless, the number of required input variables is more than doubled. For example, model 25 requires 6 input variables while model 43 requires 15 input variables for the same sensitivity. A much larger number of input variables would be more difficult to implement in clinical practice.

**Table 6:** Best models for different sensitivity levels, with a number of input variables > 12

Model	Model Type	Number of input variables	Data Augmentation	Accuracy	Sensitivity	Specificity	Recall Macro	AUC ROC	FP	FN	FP + FN
37	MLP	15	DA LE	0.3003	1	0.2042	0.6021	0.8210	226	0	226
38	SVM	15	w/o DA	0.5697	0.9744	0.5141	0.7442	0.7872	138	1	139
39	MLP	13	DA LE	0.5820	0.9487	0.5317	0.7402	0.8093	133	2	135
40	SVM	15	w/o DA	0.6099	0.9231	0.5669	0.7450	0.7872	123	3	126
41	MLP	13	w/o DA	0.6409	0.8974	0.6056	0.7515	0.8152	112	4	116
42	MLP	14	DA LE	0.7059	0.8718	0.6831	0.7774	0.7968	90	5	95
43	MLP	15	DA LE	0.7214	0.8462	0.7042	0.7752	0.7988	84	6	90
44	SVM	15	DA EO	0.7337	0.8205	0.7218	0.7712	0.8125	79	7	86
45	SVM	15	DA EO	0.7461	0.7949	0.7394	0.7672	0.8125	74	8	82

Abbreviations: *w/o DA* No data augmentation, *DA LE* Data augmentation w/limited expert range, *DA EO* Data augmentation w/expert original range

In the clinical context, one of the main focuses of the GDM specialists is the balance between sensitivity and specificity. High sensitivity avoids errors in detecting patients with the illness (low FN), while high specificity decreases the FP number. Tables 5 and 6 show a trade-off between sensitivity and specificity in our results, yielding a high, but not maximum, AUCROC. The models are ordered on Table 5, first by a sensitivity level, and then other selected metrics, such as specificity and AUCROC. The main metrics used in the final selection of our models were sensitivity and specificity. We also used a Balanced Random Forest (BRF) model that had good performance on imbalanced datasets, that achieved good performance, although not better than that of the models presented on Tables 5 and 6.

On Table S1 (Additional file 1), we show the Mean AUCROC, 95% confidence interval, and standard deviation (STD) of the different models presented on Tables 5 and 6, calculated by using ten different seeds for the initialization of the models.

Table 7 presents performance comparisons among the models with Data Augmentation (w/DA), and without Data Augmentation (w/o DA). The comparisons include the same models.

**Table 7:** Comparison of performance between models with Data Augmentation (w/DA), and without (w/o) data augmentation

Model Number	Model Type	Number of input variables	Data Augmentation	Accuracy	Sensitivity	Specificity	Recall Macro	ROC	FP	FN	FP + FN
1 w/DA	MLP	12	DA EO	0.3313	1	0.2394	0.6197	0.7505	216	0	216
1 w/o DA	MLP	12	w/o DA	0.3994	1	0.3169	0.6585	0.8189	194	0	194
5 w/DA	MLP	8	DA LE	0.5511	0.9744	0.4930	0.7337	0.8002	144	1	145
5 w/o DA	MLP	8	w/o DA	0.4303	0.9744	0.3556	0.6650	0.8172	183	1	184
9 w/DA	SVM	5	DA EO	0.6068	0.9487	0.5599	0.7543	0.8234	125	2	127
9 w/o DA	SVM	5	w/o DA	0.4396	0.9487	0.3697	0.6592	0.8221	179	2	181
13 w/DA	SVM	5	DA EO	0.6130	0.9231	0.5704	0.7468	0.8234	122	3	125
13 w/o DA	SVM	5	w/o DA	0.5913	0.9231	0.5458	0.7344	0.8221	129	3	132
17 w/DA	MLP	5	DA LE	0.6594	0.8974	0.6268	0.7621	0.8199	106	4	110
17 w/o DA	MLP	5	w/o DA	0.5944	0.8974	0.5528	0.7251	0.8202	127	4	131
25 w/DA	MLP	6	DA LE	0.7090	0.8462	0.6901	0.7681	0.8142	88	6	94
25 w/o DA	MLP	6	w/o DA	0.6099	0.8462	0.5775	0.7118	0.8156	120	6	126
29 w/DA	SVM	12	DA LE	0.7368	0.8205	0.7254	0.7729	0.8129	78	7	85
29 w/o DA	SVM	12	w/o DA	0.7554	0.8205	0.7465	0.7835	0.8135	72	7	79
33 w/DA	SVM	7	DA LE	0.7399	0.7949	0.7324	0.7636	0.8143	76	8	84
33 w/o DA	SVM^a^	7	w/o DA	0.5635	0.8205	0.5282	0.6743	0.7852	134	7	141
33 w/o DA	SVM^a^	7	w/o DA	0.6161	0.7692	0.5951	0.6822	0.7852	115	9	124

Abbreviations: *w/o DA* No data augmentation, *DA LE* Data augmentation w/limited expert range, *DA EO* Data augmentation w/expert original range

^a^ Obtained with the closest sensitivity value (validation set)

## Discussion

The principal findings of this study are: i) Early prediction of GDM within early stages of pregnancy using regular examinations/exams; ii) The development and optimization of twelve different ML models and their hyperparameters to achieve the highest prediction performance; iii) a data augmentation method is proposed to allow reaching excellent GDM prediction results with various models; and iv) several model results are, in general, better than previously reported methods generated using similar input datasets, and the models studied allow the selection of several alternatives to achieve a desired sensitivity and specificity.

A recent study by Pillay and co-workers [51] reported sensitivity and specificity data for two-step oral glucose challenge tests with 140- and 135-mg/dL at or after 24 weeks of gestation [51]; these two cut-off levels had sensitivities of 82% and 93%, respectively, and specificities of 82% and 79%, respectively, when assessed against Carpenter and Coustan criteria [51]. Interestingly, the authors also concluded that although the application of the one-step (IADPSG) protocol significantly increased the likelihood of GDM detection (11.5% vs. 4.9%; five randomised control trials, 25,772 subjects), there was no improvement in health outcomes [51]. It is possible that the use of the IADPSG protocol may be over diagnosing risk in the assessed populations and as a result the deployment of interventions to patients that would otherwise go untreated conveyed no benefit. A second interpretation is that the interventions targeted to women detected with the one-step test were ineffectual when deployed at or towards the end of the second trimester. In keeping with the potential benefit of a ML-based system allowing for earlier GDM risk prediction, it is tempting to speculate that earlier identification and intervention allocation may improve treatment benefit.

### Comparison with state of the art

In the present study, the best performing models (i.e., SVM 12; Table 5) using data collected prior to 20 weeks of gestation had a sensitivity of 82% and specificity of 74%, coming quite close to that of the two-step protocol widely used in the United States at later gestations. In our study, we developed a group of 12 models for early diagnosis of GDM, with data that are commonly acquired at the early stages of pregnancy during prenatal care visits to gynecologists/obstetricians. The ease of data collection should facilitate the future of these models in clinical practice. Another important consideration is that sensitivity is crucial since the main goal is to prevent serious consequences of GDM for mothers and babies, many of which will impact them for several years after pregnancy. In cases of lower specificity (higher FP), additional tests could be used to improve diagnosis, although this would come with additional cost, inconvenience, and risk. Also, in many cases the main treatment involves diet and exercise which are not harmful. From our variable selection methods, the most important variables for GDM diagnosis were related to glucose metabolism (first trimester fasting glycemia), physical status (weight and BMI), age, and hypertension. The use of DA had a positive effect in most models, improving specificity up to 51.43% and AUCROC up to 3.70% with the same sensitivity. The best model results, for each sensitivity level, was reached in 7/9 cases with DA and in 2/9 with no DA.

The limited public availability of datasets for informing previously published work makes direct comparisons of model performance difficult [20–35]. Nevertheless, a general assessment can be undertaken by comparing the result ranges from different metrics obtained on various datasets. However, there are important aspects, such as characteristics of the population, and diagnostic criteria, that vary between countries/regions in the different studies analyzed, and therefore, these aspects should be considered when comparing the different datasets. Table 8 shows a comparison of model results from the present study against those of recent studies assessing ML-driven diagnosis of GDM risk. In general, our models performed better in AUCROC than comparable models generated with similar input variables and the same or similar GDM diagnosis criteria [20–22, 25–28, 30, 31]. As explained previously, sensitivity is important due to the possible adverse effects of GDM on the mother and baby later in life. Other models [20, 22–24, 29, 32–35] that required additional complex data are not listed in Table 8. In some cases, such as those presented in the meta-analysis [52], more complex variables were employed on the models such as ultrasound screening data, or biochemical data of liver/renal/coagulation function at the prenatal visit. For example, a comparison between our model 33 SVM 7 Variables DA LE (Table 8), and the work of Wu and colleagues [20] (Table 8) yielded a higher sensitivity (13.55%), and a higher specificity (6.14%). Our model 17 MLP 5 Variables DA EO (Table 8) vs. Pintaudi et al*.* [28] (Table 8), reached a similar sensitivity but had an improved specificity (56.70%). A different criterion for GDM diagnosis was by Kumar and coworkers [31] (WHO, 1999), with which GDM was diagnosed if fasting OGTT ≥ 126 mg/dL and/or 140 mg/dL in a 2 h OGTT. Another model was implemented by them [31] using the same GDM diagnosis criterion as ours, IADPSG/HAPO, reaching an AUCROC of 0.73, with a fivefold stratified CV. ML models have also been applied for predicting Diabetes Mellitus [53].

**Table 8:** Results of top models for various levels of sensitivity compared to those from the published literature using similar input variables and the same GDM diagnosis criterion

Models	Accuracy	Sensitivity	Specificity	Recall Macro	AUC ROC
DNN, 7 Variables [20]	-	0.7	0.69	0.695*	0.77
LR, 5 Continuous Variables [21]	-	0.61	0.80	0.705*	0.766
LGBM, 9 questions (Variables) [22]	-	-	-	-	0.799
RF, Dimension Reduction, 6 Variables [25]	0.789	0.651	0.813	0.732*	0.777
LR, 4 Variables [26]	-	-	-	-	0.70
1 Variable ** [27]	-	0.490	0.676	0.583*	0.608
RECPAM, 3 Variables [28]	-	0.89	0.40	0.645*	-
2 Variables ** [30]	-	0.51	0.81	0.660*	0.71
NN, 4 Variables, IADPSG Criteria [31]	-	-	-	-	0.73
Ours 1 MLP 12 Variables No DA	0.3994	1	0.3169	0.6585	0.8189
Ours 5 MLP 8 Variables DA LE	0.5511	0.9744	0.4930	0.7337	0.8002
Ours 9 SVM 5 Variables DA EO	0.6068	0.9487	0.5599	0.7543	0.8234
Ours 13 SVM 5 Variables DA EO	0.6130	0.9231	0.5704	0.7468	0.8234
Ours 17 MLP 5 Variables DA EO	0.6594	0.8974	0.6268	0.7621	0.8199
Ours 21 MLP 7 Variables DA LE	0.6873	0.8718	0.6620	0.7669	0.8160
Ours 25 MLP 6 Variables DA LE	0.7090	0.8462	0.6901	0.7681	0.8142
Ours 29 SVM 12 Variables No DA	0.7554	0.8205	0.7465	0.7835	0.8135
Ours 33 SVM 7 Variables DA LE	0.7399	0.7949	0.7324	0.7636	0.8143

^*^Values calculated by us from the results displayed, using the formula of recall macro (sensitivity + specificity)/2

^**^Deterministic Model

Ours (model number-Table 5)

Note: Datasets used in some previous studies are different and not publicly available

Table 9 shows a list of the input variables used in each of the best models selected, including those used for comparison, and those developed and selected by the authors. It can be observed that some of the best solutions require only five input variables. When choosing these models for a clinical application, only 5–7 variables will need to be measured in each patient to diagnose GDM with these models. This will facilitate the possible application of these models in clinical practice. Developing accurate ML models for predicting GDM is an important step towards implementing early prediction and treatment strategies for patients. The next step should be to prospectively apply them in a clinical setting to validate and evaluate their performance.

**Table 9:** Input variables used in each model including those used for comparison, and those of the best models selected by our method

Models	Input Variables
DNN, 7 Variables [20]	Age, Previous GDM, Family history of diabetes in a first-degree relative, Multiple pregnancy, FPG, HBA_1C_, Triglyceride
LR, 5 Continuous Variables [21]	Age, pre-pregnancy BMI, FPG and Triglyceride
LGBM, 9 questions (Variables) [22]	Age, Weight and Height, Familiar history of diabetes in first-degree relatives, High cholesterol, Miscarriage, PCOS, Pre-diabetes, Heart Diseases, GDM or High BP before current pregnancy, HBA_1C_, Previous birth (Yes or No), if yes, number of times and GCT or OGTT in that pregnancy if they are available
RF, Dimension Reduction, 6 Variables [25]	Age, pre-pregnancy BMI, abdomen circumference in the first trimester, gravidity, PCOS, irregular menstruation and family history of diabetes
LR, 4 Variables [26]	Age, BMI, FPG, Familiar history of diabetes in first-degree relatives
1 Variable * [27]	FPG
RECPAM, 3 Variables [28]	BMI, FPG, Familiar history of diabetes in first-degree relatives
2 Variables * [30]	BMI, fasting glucose
NN, 4 Variables, IADPSG Criteria [31]	Mean arterial blood pressure, Age, Previous history of GDM, Ethnicity
Ours 1 MLP 12 Variables No DA	Age, Weight, BMI, Illicit Drugs, Cardiac Diseases, Urinal Tract Diseases, Psychiatric Disorders, Chronic Kidney Diseases, Inflammatory bowel disease, Insulin Resistance, Use of Antihypertensive drugs, FPG
Ours 5 MLP 8 Variables DA LE	Age, Weight, BMI, Illicit Drugs, Chronic Kidney Diseases, Inflammatory bowel disease, Use of Antihypertensive drugs, FPG
Ours 9 SVM 5 Variables DA EO	Age, Weight, BMI, Gravidity, FPG
Ours 13 SVM 5 Variables DA EO	Age, Weight, BMI, Gravidity, FPG
Ours 17 MLP 5 Variables DA EO	Age, Weight, BMI, Gravidity, FPG
Ours 21 MLP 7 Variables DA LE	Age, Weight, BMI, Gravidity, Parity, Chronic Hypertension, FPG
Ours 25 MLP 6 Variables DA LE	Age, Weight, BMI, Inflammatory bowel disease, Use of Antihypertensive drugs, FPG
Ours 29 SVM 12 Variables No DA	Age, Weight, BMI, Illicit Drugs, Cardiac Diseases, Urinal Tract Diseases, Psychiatric Disorders, Chronic Kidney Diseases, Inflammatory bowel disease, Insulin Resistance, Use of Antihypertensive drugs, FPG
Ours 33 SVM 7 Variables DA LE	Age, Weight, BMI, Gravidity, Chronic Hypertension, Use of Antihypertensive drugs, FPG

^*^Deterministic model

Ours (model number from Table 5)

In the present study, twelve ML models and their hyperparameters were optimized for early (20 weeks of gestation or earlier) GDM with high sensitivity, specificity, AUCROC, and Recall Macro. The models could predict GDM with a good degree of accuracy before 20 weeks of gestation, and with variables that are widely available from screening examinations The variables required by most of the models were age, weight, BMI, and FPG which is consistent with previous publications [20–22, 25–28, 30, 31]. Variable selection was performed by three methods and results show that several models reached good performance with as few as 5–7 input variables, while other models required more, including up to 12 variables. Choosing models with high GDM prediction performance, a low number of input variables, and widely available variables will facilitate the possible application of these models in low income settings. Although patient data from previous publications are often not available, comparing the results obtained for various metrics show that, in general, our models performed favorably in comparison with the existing literature. In conclusion, our data demonstrate that ML-analysis of patient data sets from early pregnancy may serve as a cost-effective and efficacious means of detecting GDM risk early in pregnancy.

We described all steps required to implement, train and test the models. In particular, we used a test partition that is different from the training and validation partitions, to improve the generalization capacity of the models. Many of the previous reported work did not state explicitly using an independent partition for testing [20, 21, 24–32, 35]. This study provides a valuable contribution by utilizing and comparing a broad range of ML models (12), which differs from many other studies that often use only one type of model, such as Logistic Regression. Additionally, various metrics have been employed to compare the performance of each model, including a wide range of variables that could potentially be selected for clinical implementation. This approach allows for a more comprehensive assessment of the potential utility of different ML models in predicting GDM and facilitates the identification of the most effective models for future clinical implementation.

As with any study of this nature, the findings need to be assessed in light of the ground-truth data set from which they were drawn. For the present study, we used a single center population drawn from a socio-economically vulnerable medical center in Santiago, Chile. Accordingly, a cautious approach should be taken in extrapolating these findings to a wider socio-economic grouping, and to the maternal situation in other regions. The strengths of this study include a well-characterized pregnancy cohort and robust data collection. Future iterations of this work will involve the cross-population analysis of GDM risk and the comparison of predictive outcomes from different populations to assess the broad applicability of model performance. While the variables used in the different ML models show promising predictive capacity for GDM, the addition of other inputs such as biomarkers could potentially further improve their performance. As such, future studies may consider incorporating additional data sources to enhance the accuracy of GDM prediction models.

These findings are of particular importance given the increasing prevalence of GDM in the maternal population and the significant impacts (both patient well-being and financial) that derive from poorly controlled glucose levels in pregnancy. For example, recent modeling from the United States suggests that, in 2014, the short-term costs of GDM were $1.8 billion [54]. The cost of treatment for T2DM is routinely around $3,500 per year [55]. Given estimates that one in six pregnancies are impacted by GDM, even a small improvement in outcomes deriving from early risk identification and timely intervention would yield profound public health benefits and health system cost savings.

## Conclusions

The principal findings of our study are: Early prediction of GDM within early stages of pregnancy using regular examinations/exams; the development and optimization of twelve different ML models and their hyperparameters to achieve the highest prediction performance; a novel data augmentation method is proposed to allow reaching excellent GDM prediction results with various models. Several model results are, in general, better than previously reported methods generated using similar input datasets, and the provided results allow the selection of several alternatives to achieve a desired sensitivity and specificity. Choosing models with high GDM prediction performance, a low number of input variables, and widely available variables will facilitate the possible application of these models in most settings.

## Supplementary Information

**Additional file 1:** **Table S1.** The Mean AUCROC bracketed values are at the 95% confidence interval, and standard deviation (STD) of the different models presented in Tables 5 and 6. STD uses four decimals.

## Data Availability

The datasets used in this study are not publicly available due to privacy reasons. The dataset is provided by Hospital Parroquial de San Bernando, access to this data may be provided to qualified researchers upon request and permission of this institution (sillanes@uandes.cl). The code used for the analysis, data cleaning, and model implementation are not available due to proprietary reasons and requires the data to be used. The models were implemented with Python using libraries which are publicly available for anyone that wants to replicate the experiments.

## Abbreviations

1TFG: First trimester fasting glucose test
ANOVA: Analysis of variance
AUCROC: Area under of curve of receiver operating characteristic
BMI: Body mass index
BNB: Bernoulli Naïve Bayes
BRF: Balanced random forest
CV: Cross validation
DA: Data augmentation
DNN: Deep neural network
DT: Decision tree
ET: Extra trees
FN: False negative
FPG: Fasting plasma glucose
FP: False positive
GB: Gradient boosting
GDM: Gestational diabetes mellitus
GNB: Gaussian Naïve Bayes
HAPO: Hyperglycemia and adverse pregnancy outcome
HBA1c: Glycated hemoglobin
IADPSG: International association of diabetes and pregnancy study groups
KNN: K-nearest neighbors
LGBM: Light gradient boosting
LR: Logistic regression
ML: Machine learning
MLP: Multi-layer perceptron
OGTT: Oral glucose tolerance test
RF: Random forest
SVM: Support vector machine
T2DM: Type 2 diabetes mellitus
WHO: World health organization
XGB: Extreme gradient boosting
