# Supplementary material for: Development of machine learning models to predict gestational diabetes risk in the first half of pregnancy
Source: BMC Pregnancy Childbirth. 2023 Jun 23;23:469. doi: 10.1186/s12884-023-05766-4 (PMC10288662; doi:10.1186/s12884-023-05766-4)
Supplement: Supplementary file 1 — Additional file 1: Table S1. The Mean AUCROC bracketed values are at the 95% confidence interval, and standard deviation (STD) of the different models presented in Tables 5 and 6. STD uses four decimals. [file 12884_2023_5766_MOESM1_ESM.docx]

**Table S1**: The Mean AUCROC bracketed values are at the 95% confidence interval, and standard deviation (STD) of the different models presented in Tables 5 and 6. STD uses four decimals.

| **Model Number** | **AUCROC** | **Standard Deviation** |
| --- | --- | --- |
| **1** | **0.8157 [0.8120, 0.8193]** | **0.0052** |
| 2 | 0.7778 [0.7640, 0.7915] | 0.0192 |
| 3 | 0.7158 [0.6728, 0.7589] | 0.0602 |
| 4 | 0.7144 [0.6654, 0.7633] | 0.0684 |
| **5** | **0.8099 [0.8064, 0.8133]** | **0.0049** |
| 6 | 0.8161 [0.8160, 0.8161] | 0.0000 |
| 7 | 0.8161 [0.8160, 0.8161] | 0.0000 |
| 8 | 0.8007 [0.7980, 0.8034] | 0.0038 |
| **9** | **0.8234 [0.8234, 0.8234]** | **0.0000** |
| 10 | 0.8156 [0.8151, 0.8161] | 0.0007 |
| 11 | 0.8173 [0.8167, 0.8178] | 0.0008 |
| 12 | 0.7178 [0.5968, 0.8389] | 0.1692 |
| **13** | **0.8234 [0.8234, 0.8234]** | **0.0000** |
| 14 | 0.8193 [0.8174, 0.8211] | 0.0026 |
| 15 | 0.8174 [0.8161, 0.8187] | 0.0019 |
| 16 | 0.8159 [0.8159, 0.8159] | 0.0000 |
| **17** | **0.8104 [0.8040, 0.8167]** | **0.0089** |
| 18 | 0.8095 [0.8051, 0.8140] | 0.0062 |
| 19 | 0.8141 [0.8123, 0.8158] | 0.0025 |
| 20 | 0.8102 [0.8081, 0.8123] | 0.0030 |
| **21** | **0.8089 [0.8030, 0.8147]** | **0.0082** |
| 22 | 0.8047 [0.7912, 0.8182] | 0.0189 |
| 23 | 0.8113 [0.8090, 0.8135] | 0.0031 |
| 24 | 0.8121 [0.8104, 0.8139] | 0.0024 |
| **25** | **0.8162 [0.8152, 0.8172]** | **0.0014** |
| 26 | 0.7972 [0.7875, 0.8069] | 0.0135 |
| 27 | 0.8081 [0.8067, 0.8095] | 0.0020 |
| 28 | 0.7972 [0.7875, 0.8069] | 0.0135 |
| **29** | **0.8135 [0.8135, 0.8135]** | **0.0000** |
| 30 | 0.8135 [0.8135, 0.8135] | 0.0000 |
| 31 | 0.8143 [0.8142, 0.8143] | 0.0001 |
| 32 | 0.8143 [0.8142, 0.8143] | 0.0001 |
| **33** | **0.8143 [0.8142, 0.8143]** | **0.0001** |
| 34 | 0.7538 [0.6103, 0.8974] | 0.2007 |
| 35 | 0.6961 [0.6558, 0.7363] | 0.0562 |
| 36 | 0.7487 [0.6902, 0.8072] | 0.0818 |
| 37 | 0.8091 [0.8047, 0.8134] | 0.0061 |
| 38 | 0.7872 [0.7872, 0.7872] | 0.0000 |
| 39 | 0.7917 [0.7791, 0.8043] | 0.0018 |
| 40 | 0.7872 [0.7872, 0.7872] | 0.0000 |
| 41 | 0.8150 [0.8134, 0.8166] | 0.0022 |
| 42 | 0.7163 [0.6477, 0.7849] | 0.0959 |
| 43 | 0.7827 [0.7640, 0.8015] | 0.0262 |
| 44 | 0.8125 [0.8124, 0.8125] | 0.0000 |
| 45 | 0.8125 [0.8124, 0.8125] | 0.0000 |
